# Influence of age on speech-in-noise and spatial processing abilities in middle-aged adults

**DOI:** 10.1371/journal.pone.0341169

**Published:** 2026-01-29

**Authors:** Pavithra Nayak, Suresh Pillai, Hari Prakash Palaniswamy

**Affiliations:** 1 Department of Speech and Hearing, Manipal College of Health Professions, Manipal Academy of Higher Education, Manipal, India; 2 Department of Head and Neck Surgery, Kasturba Medical College, Manipal Academy of Higher Education, Manipal, India; Father Muller Charitable Institutions, INDIA

## Abstract

Speech perception in noise (SPIN) difficulties are commonly associated with older adults, but emerging evidence suggests they may begin in midlife. This study investigated SPIN and spatial processing abilities in middle-aged adults using the Spatial Separation Sentence Test-Kannada (SSST-K). A cross-sectional design assessed 76 participants aged 41–60 years, divided into two groups (41–50 and 51–60 years). Assessments included: (1) the SSST-K to evaluate SPIN and spatial processing, (2) the Montreal Cognitive Assessment (MoCA) for general cognition, and (3) standardized questionnaires measuring noise exposure (NESI), physical activity (GPAQ), and mental status (PHQ-9). Hierarchical regression analysis revealed two key findings. First, in the co-located (0° azimuth) condition, cognition (MoCA) was the only significant predictor of SPIN performance, overshadowing age and other variables. Second, none of the tested variables predicted spatial advantage. Between-group comparisons showed no age-related differences in SPIN scores for the co-located condition, but the older subgroup (51–60 years) exhibited reduced spatial advantage and significantly lower MoCA scores. These results indicate that among the variables examined in middle-aged adults with self-reported normal hearing, cognitive function emerged as the strongest predictor of SPIN ability. However, the absence of audiometric verification, particularly extended high-frequency assessment, limits definitive conclusions about peripheral versus central contributions. The other variables, like noise exposure, mental health, and physical activity, had no significant influence. Future research with longitudinal designs and objective hearing assessment is needed to determine optimal ages and methods for SPIN screening in community and clinical settings.

## 1. Introduction

Age-related hearing loss is a common health issue, particularly as the global population ages. It is well established that the gradual decline in auditory function significantly contributes to the complex speech perception challenges faced by older adults [1–3]. Even individuals with audiometrically normal hearing may encounter difficulties understanding speech, especially in environments with background noise [4,5].

Studies suggest that individuals may start experiencing difficulties in understanding speech in noisy settings as early as middle age. Specifically, difficulties in hearing speech against background noise often appear between the ages of 40 and 65 [6–8]. McFarlane and Sanchez [9] found that middle-aged adults (40–60 years) with normal hearing thresholds often demonstrate reduced SPIN performance, suggesting that subtle central auditory processing deficits may explain real-world communication challenges in this population. A recent review highlighted that middle-aged adults often exhibit increased listening effort and reduced speech perception in noise compared to younger adults [10].

Numerous factors may influence speech-in-noise performance among middle-aged adults. Emerging evidence suggests that specific types of noise exposure, particularly occupational noise, may contribute to difficulties in speech perception [11,12]. Cognitive abilities play a significant role in speech perception in noise (SPIN) performance among adults [13]. The level of education also greatly impacts auditory processing and the ability to understand speech in noisy situations among middle-aged and older adults. Individuals with higher education levels generally exhibit superior speech comprehension in these environments, possibly because of a strengthened cognitive reserve that helps them cope with degraded auditory input [14].

Psychological factors such as depression may also significantly impact SPIN performance. Xie et al. [15] examined the impact of major depressive disorder (MDD) on speech perception in noise among normal-hearing adults. Their findings indicated that individuals with MDD demonstrated significantly lower recognition accuracy, particularly in conditions with a single-talker masker. The depression-related deficit in speech perception was specific to errors resulting from interference by masker sentences, suggesting heightened distractibility due to linguistic interference from background talkers.

The relationship between physical activity and SPIN remains uncertain. Jiang et al. [16] found that SPIN performance, measured using the QuickSIN, predicted cognitive decline independently of physical activity levels, suggesting that exercise may not have a strong direct or indirect effect on SPIN. In contrast, Gopinath et al. [17] reported that practicing Sudarshan Kriya Yoga improved specific auditory processing skills, such as gap detection and pitch discrimination thresholds, although improvements in SPIN were inconclusive. Together, these findings suggest that while physical activity may enhance certain auditory processing abilities, its impact on SPIN performance may not be straightforward.

Existing literature has examined SPIN performance using co-located noise conditions, where speech and noise sources originate from the same spatial location. However, real-life listening environments typically involve spatially separated sound sources, which enable listeners to utilize binaural cues for improved speech understanding. Spatial separation of speech and noise can provide a substantial advantage in speech recognition, often referred to as spatial release from masking or spatial advantage. Understanding how middle-aged adults perform under spatially separated listening conditions is therefore critical for ecological validity and clinical relevance.

The Spatial Separation Sentence Test (SSST-K) was developed to assess speech perception abilities under spatially separated speech and noise conditions in Kannada-speaking populations [18]. Given the increasing recognition that spatial processing abilities may decline with age and may be affected by factors beyond peripheral hearing sensitivity, such as cognition [19,20]. and lifestyle [21,22]. Validating the test’s sensitivity to age-related and individual differences in this population is essential. Such validation would enable clinicians to better identify individuals who experience speech perception difficulties in real-world listening environments despite having clinically normal audiograms.

Together, these findings emphasize the need for further investigation into factors affecting speech perception in noise (SPIN) among middle-aged adults. Prior validation work with the SSST-K has focused on children aged 8–12 years, showing good psychometric properties and age sensitivity in pediatric populations. Although age-related declines in speech-in-noise performance have been demonstrated with a variety of English language tests, there is a paucity of data on spatial hearing in Kannada-speaking middle-aged adults. The present study therefore aimed (i) to evaluate the sensitivity of the SSST-K to age-related changes in spatial processing among adults aged 41–60 years, and (ii) to examine the relative contributions of cognition, noise exposure, mood, and physical activity to SSST-K performance in a community-based, Kannada-speaking cohort.

## 2. Materials and methods

### 2.1. Participants

Seventy-six participants aged between 41 and 60 years were recruited for the study, divided into two groups: 38 individuals aged 41–50 years and 38 individuals aged 51–60 years, ensuring equal distribution of male and female participants. All participants were native Kannada speakers, and the sample was obtained through the snowball sampling method.

The age cutoff of 50 years was selected based on converging evidence from the literature. Study by Moore et al. and Banumathi et al. [12,23] identified the fifth decade as a critical period for emerging speech-in-noise difficulties. Additionally, Stelmachowicz et al. [24] reported that the largest age-related changes in EHF sensitivity occur between 40 and 59 years, suggesting a potential inflection point around 50. This 10-year grouping also provides sufficient statistical power while capturing meaningful developmental changes across middle adulthood.

The study included middle-aged adults with self-reported normal hearing, as it was the practical screening approach in community-based studies. Self-reported hearing status has shown moderate correlation with audiometric thresholds in population studies [25,26]. Individuals with cardiovascular diseases, chronic medical conditions, or pre-existing neurological and psychological disorders were excluded from participation.

### 2.2. Procedure

Study approval was obtained from the Institutional Research and Ethical Committee (IEC2: 383/2024). Data were collected from participants residing in Udupi, Karnataka, from November 6, 2024, to March 17, 2025. The procedure was briefly explained to the participants, and written informed consent was obtained from all participants prior to data collection. The participant’s case history was received, including demographic details like age, gender, education, work, History of hearing loss and/or speech understanding difficulty, otological, medical, and surgical history, along with details on workplace noise exposure and the use of PLDs.

Then, all the assessments were conducted individually in a quiet, well-lit room to avoid distractions and get an accurate response. The assessment was conducted in a fixed order: MoCA first, followed by PHQ-9, NESI, GPAQ, and SSST-K. The entire procedure took around 30–45 minutes for each participant.

### Montreal cognitive assessment tool (MoCA)

MoCA, developed by Nasreddine et al. [27] was administered in its validated Kannada adaptation [28] adaptation to ensure linguistic and cultural appropriateness.. This 10-minute screening tool assesses visuospatial/executive abilities, naming, short-term memory, attention, language, abstraction, delayed recall, and orientation domains. The maximum score is 30, with scores ≤ 26 indicating cognitive impairment. Participants with less than or equal to 12 years of education received an extra point per MoCA guidelines to account for educational bias. The Kannada MoCA was validated as part of a multi-language study [29] that included 61 Kannada speaking cognitively healthy controls and 39 individuals with mild cognitive impairment (MCI), demonstrating 86% sensitivity and 89% specificity for detecting MCI against clinical diagnostic standard. The test was conducted face to face by the tester following the standard instructions.

### Patient health questionnaire – 9 (PHQ-9)

PHQ-9, developed by [30], is a 9-item self-reported tool that assesses depression severity over the preceding two weeks. Each is scored from 0 (“not at all”) to 3 (“nearly every day”), with total scores that range from 2 to 27. In this study, we used the Kannada-translated version of the PHQ-9, which has been validated for use in the Indian population [31]. The validity of the Kannada version is comparable with the original version. It showed a sensitivity of 86.7% and specificity of 88.5% for detecting major depressive disorder [31]. Severity is classified into the following categories: minimal (0–4), mild [5–9], moderate [10,12–15], moderately severe [16–20], and severe [21–28].

### Noise exposure structured interview (NESI)

NESI, developed by Guest et al. [32] is a validated tool for quantifying lifetime noise exposure. It is a structured questionnaire that collects information about an individual’s exposure to loud sounds in different recreational, occupational, and educational settings.

This interviewer-administered tool collects detailed information about exposure duration (in years, weeks/year, and hours/week), intensity (categorized into 5 dB bands from <80 dBA to ≥95 dBA), and frequency from various sources, such as workplace noise, loud music, and firearms. The cumulative noise exposure is calculated in A-weighted decibels (dBA) using a standardized formula: Units of noise exposure = [(years × weeks × days × hours)/ 2080] x 10^[(L-90)/10] for each reported exposure, where L is the sound level in dBA [32]. The total exposure is derived by combining values across reported activities.

The NESI demonstrates robust psychometric properties, with good test-retest reliability in validation studies. In the current study, the tester administers the NESI in a face-to-face interview format (duration~10 minutes), ensuring consistent interpretation of exposure categories and accurate data collection for all participants.

### Global physical activity questionnaire (GPAQ)

GPAQ version 2, given by the World Health Organization (WHO), is a validated 16-item instrument assessing physical activity patterns across three domains: (1) work-related activities, (2) transportation, and (3) recreational pursuits. The questionnaire captures activity intensity (Vigorous/moderate), frequency (days/week), duration (minutes/day) for each domain, and sedentary behavior. Metabolic equivalent of task (MET) minutes per week is calculated using preset values (8.0 METs for vigorous activity, 4.0 METs for moderate activity) multiplied by duration. Participants are classified into activity levels based on their total MET minutes per week: low activity is defined as less than 600 minutes, moderate activity ranges from 600 to 3000 minutes, and high activity exceeds 3000 minutes.

Armstrong and Bull [33] (10 described the development and initial implementation of the GPAQ, while Bull et al. [34] provided the comprehensive validation of the instrument, demonstrating moderate to good test-retest reliability (kappa statistics of 0.67 to 0.73) and concurrent validity coefficients of 0.54. For this study, the GPAQ was administered orally in Kannada through face-to-face interviews, with the interviewer translating questions directly to ensure participants comprehension while maintaining fidelity to the original questionnaire structure.

### Spatial separation sentence test – Kannada

Spatial Separation Sentence test (SSST) is a validated MATLAB-based auditory assessment tool developed by Hermon et al. [18,35] to evaluate speech in noise perception in native Kannada speakers. A 2.5 dB up-and-down procedure was used to present the stimulus at various SNRs, and the participants were asked to listen to sentences attentively while ignoring the distractor and repeat all the sentences they had heard. They were allowed to guess the words when they were not sure. The response was considered correct only if the participant was able to accurately repeat all the content words in the sentence. Upon achieving a 50% score at an SNR, the SNR was reduced by 2.5 dB. The ceiling was reached if the participant responded less than 50% after three consecutive presentations. This level of SNR was taken as the client’s SNR for that test condition (0°, + 90°, and −90°).

SSST-K was administered using a custom MATLAB-based script provided by the original developer. Stimuli were presented through JBL Quantum 100 wired over-ear headphones. Calibration was performed to ensure the output sound pressure level (SPL) matched the original SSST-K specifications. The headphones were calibrated with an NBS-9A coupler using a Brüel & Kjær type 2250 Light sound level meter to verify if the SPL of the stimuli ranged between 68- and 72-dB SPL at 20% system output.

In the present study, the JBL headphones were preferred because they are an affordable, widely available model with adequate bandwidth (20 Hz–20 kHz) and sensitivity, and all testing was conducted after level calibration to match the SSST K presentation requirements. Importantly, the same headset was used for every participant, which preserves internal validity and allows for a reliable estimation of relative SNR 50 differences within the study, even if absolute thresholds may not be directly comparable to those in studies using other transducers.

### 2.3. Statistical analysis

Data obtained through the demographic data, questionnaire, and SSST-K were entered into an Excel sheet and transformed into JAMOVI (Version 2.6.44). Descriptive statistics were computed for all variables. The normal distribution of the data was evaluated using the Shapiro-Wilk test. Due to the data not being normally distributed, a non-parametric approach was utilized, specifically the Mann-Whitney U test, to compare SSST-K scores across two age groups: 41–50 years and 51–60 years.

Spearman’s rank order correlation was used to explore the associations between spatial advantage and 0-degree azimuth condition scores and each covariate, such as age, education level, NESI, MoCA, PHQ-9, and GPAQ. A hierarchical multiple linear regression was then conducted separately for spatial advantage and 0-degree condition outcomes to determine the influence of covariates.

Collinearity statistics revealed acceptable VIF values (all VIF < 2.0), indicating no multicollinearity. Shapiro-Wilk test of residuals indicated marginal deviation from normality (W = 0.966, p = .010); however, the Q-Q plot of residuals revealed that residuals were approximately normally distributed, with minor deviations at the extremes. The residual plot demonstrated homogeneous variance across fitted values, confirming the homoscedasticity assumption. Cook’s distance analysis identified no influential outliers. The maximum Cook’s distance was 0.0975 (well below the conventional threshold of 1.0), with a median of 0.0175 and a mean of 0.00454, indicating that all observations had minimal and comparable influence on the regression results. These findings align with established guidance that linear regression is robust to minor deviations from normality.

The models were built in a stepwise manner as follows:

Model 1: Age
Model 2: Age + MoCA
Model 3: Age + MoCA + Education
Model 4: Age + MoCA + Education + NESI
Model 5: Age + MoCA + Education + NESI + PHQ-9
Model 6: Age + MoCA + Education + NESI + PHQ-9 + GPAQ

The analysis adhered to a significant level of p < .05. Multicollinearity was evaluated using the Variation Inflation Factor (VIF) and tolerance values for all predictors in each regression model. A conservative VIF threshold of <2.5 was used to indicate acceptable multicollinearity. All variables in all models had VIF values well below this threshold (see S1 File), indicating that multicollinearity was not a concern and all predictors could be retained in the models.

## 3. Results

### 3.1. Demographics

This study collected data from 76 middle-aged adults, equally divided into two age groups: 41–50 years (n = 38) and 51–60 years (n = 38). Each group included an equal number of males and females (19 males and 19 females). Given the non-normal distribution of most variables (Shapiro-Wilk p < .05), all data are presented as median (interquartile range), and non-parametric Mann-Whitney U tests were used for group comparisons. **Table 1** summarizes demographic details, including age, gender distribution, and years of education across the two age groups.

**Table 1:** Participant demographics by age group.

Variables	41-50 years (n = 38)	51-60 years (n = 38)
**Age, Median (IQR)**	45 (4.8)	53.5 (6)
**Gender, n%**	Male: 19 (50%)	Male: 19 (50%)
	Female: 19 (50%)	Female: 19 (50%)
**Education, Median (IQR)**	12 (5)	10 (6.5)

*Note. Data are presented as median (interquartile range) due to non-normal distributions.*

### 3.2. Comparison between age groups

**Fig 1:**
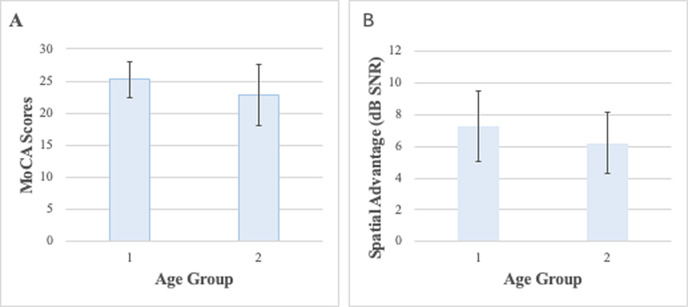
Between-group comparison of MoCA and spatial advantage scores in middle-aged adults. **(A**) MoCA total scores (0–30) by age group. Higher scores indicate better cognitive functioning. Displays cognitive scores in adults aged 41–50 and 51–60 years. Higher scores indicate better cognitive functioning. **(B)** Spatial advantage scores (dB SNR) by age group. Higher values represent greater spatial processing advantage in noisy listening environments.

Comparison between mean education, cognitive function, noise exposure, depressive symptoms, physical activity, and speech-in-noise performance is presented in **Table 2**. There were notable differences among the groups in three key areas. Education levels were higher in the younger group to the older group (p =.011, r_p_b = -.34). Cognitive function as measured by MoCA was significantly better in the 41-50 age group than in the 51-60 age group (p =.031, r_p_b = -.29). Most notably for the primary research focus, spatial advantage scores on the SSST-K were significantly higher in the younger group compared to the older group (p =.031, r_p_b = -.28), suggesting better spatial processing abilities in participants aged 41-50 years as shown in **Fig 1**. No significant between-group differences were observed for noise exposure (p =.681), depressive symptoms (p =.374), physical activity levels (p =.178), or performance in the 0° azimuth condition (p =.823).

**Table 2:** Group comparison across the key variables and SSST-K.

Variables	41-50 years (n = 38)	51-60 years (n = 38)	95% CI	*p*-value	Effect size (r_p_b)
**Education**	12 (5)	10 (6.5)	(0.00, 5.00)	**0.011**	−0.34
**MoCA**	26 (4.5)	24 (5.8)	(0.00, 4.00)	**0.031**	−0.29
**NESI**	0.28 (1.08)	0.24 (2.03)	(−0.20, 0.12)	0.681	0.06
**PHQ-9**	4 (5.75)	4 (5.75)	(−2.00, 1.00)	0.374	0.12
**GPAQ**	64.5 (139)	155 (158)	(−107.0, 11.3)	0.178	0.18
**SSST-K (0° azimuth)**	0 (5)	0 (5)	(−2.50, 0.00)	0.823	0.03
**SSST-K (Spatial advantage)**	6.88 (2.5)	6.25 (2.5)	(0.00, 2.50)	**0.031**	−0.28

*Note. All values are median (interquartile range). 95% CI = 95% confidence interval for the Hodges-Lehmann estimate of median difference (Group 1 – Group 2). CIs excluding zero indicate statistically significant differences. Bolded p-values indicate statistical significance (p < .05). MoCA = Montreal Cognitive Assessment; NESI = Noise Exposure Structured Interview; PHQ-9 = Patient Health questionnaire-9; GPAQ = Global Physical Activity Questionnaire; SSST-K = Spatial Separation Sentence Test.*

An examination of the PHQ-9 categorical distribution revealed that the majority of participants reported minimal depressive symptoms. Across the total sample, 44 participants (57.9%) scored in the minimal range (0-4), 24 (31.6%) in the mild range (5 –9 ), 8 (10.5%) in the moderate range (10 –14 ), and none reported moderately severe to severe symptoms (≥15). The categorical distribution was similar across age groups, with both groups predominantly showing minimal to mild symptoms, consistent with the non-significant median comparison (p =.374).

### 3.3. SSST- K and predictors

Spearman’s rank-order correlation analysis was carried out to examine the relationships among the primary test variables and co-variables. Performance in the 0° azimuth condition was negatively correlated with education (r = −.26, p = .022) and MoCA scores (r = −.37, p = .001). Education was significantly positively correlated with MOCA scores (r = .485, p < .001) and negatively correlated with age (r = −.39, p < .001). MoCA scores were negatively correlated with age (r = −.39, p < .001) and GPAQ (r = −.23, p = .049) and positively correlated with PHQ-9 (r = .26, p.021)). A significant negative correlation was found between education and GPAQ (r = −.23, p = .048), and a significant positive correlation was observed between NESI scores and GPAQ. (r = .29, p = .010). Spatial advantage was not significantly correlated with any of the other tested variables, including age (r = −.20, p = .077). Full correlation coefficients are presented in **Table 3**.

**Table 3:** Spearman’s rank-order correlation among key variables and SSST-K.

Variable	1	2	3	4	5	6	7	8
**0° azimuth**	–							
**Spatial advantage**	.45***	–						
**Age**	.03	−.20	–					
**Education**	−.26*	.18	−.39***	–				
**MoCA**	−.37**	−.03	−.39***	.49***	–			
**NESI**	.10	.05	.09	−.11	−.08	–		
**PHQ-9**	−.06	−.12	−.03	−.06	.26*	.21	–	
**GPAQ**	.17	.01	.19	−.23*	−.23*	.29*	.04	–

Note. *p < .05, **p < .01, ***p < .001.

### 3.4. Speech in noise performance and age

#### 3.4.1. Regression analysis: 0° azimuth.

***Primary analysis: Age as a categorical predictor:*** A hierarchical linear regression analysis (N = 76) was performed to explore the predictors of auditory performance at 0° azimuth across six consecutive models. The baseline model (Model 1), which included only age group as a predictor, showed no value (R^2^ = .0002, F(1,74) = 0.01, p = .915). Model 2 significantly improved prediction by adding MoCA scores (∆R^2^ = .148, F(1,73) = 12.68, p < .001), accounting for 14.8% of variance (b = −0.26, p < .001). Subsequent models introduced: education (Model 3; ∆R^2^ = .010, p = .363), noise exposure (Model 4; ∆R^2^ = .004, p = .576), depression symptoms (Model 5: ∆R^2^ = .002, p = .709), and physical activity (Model 6: ∆R^2^ = .003, p = .598), none providing significant improvements. The final comprehensive model (Model 6), which included all examined predictors, explained approximately 16.7% of the variance in the auditory performance, with MoCA scores remaining the only statistically significant predictor (b = −0.22, p = .022).

### Supplementary analysis: Age as a continuous predictor

For comparative purposes, we also examined age as a continuous predictor in a simple linear regression model. The model was not statistically significant, (F1, 74) =.005, p = .942). These results confirm that age, whether treated as a continuous or categorical variable, does not predict performance in the 0° azimuth condition, suggesting that co-located speech-in-noise performance in middle age is more strongly influenced by cognitive function than by age. (see **Table 4** for more details)

**Table 4:** Linear Regression Model for 0° Azimuth with Age as Continuous Predictor.

Model	R	R^2^	Overall Model Test			
			F	df1	df2	p
1	.008	<.001	.005	1	74	.942

*Note. Models estimated using a sample size of N = 76.*

#### 3.4.2. Regression Analysis: 3.4.2 Regression Analysis: Spatial advantage.

***Primary analysis: Age as a Categorical Predictor:*** A similar hierarchical regression was conducted for spatial advantage scores. Model 1 included only age group as a predictor, was a significant model (R2 = .061, F(1,74) = 4.78, p = .032), indicating that age group alone accounted for 6.1% of the variance in spatial advantage. The addition of MoCA scores in Model 2 did not result in a statistically significant improvement (∆R^2^ = .002, F(1,73) = 0.18, p = .673). Education was added to Model 3, accounting for a small, non-significant increase in explained variance (∆R^2^ = .027, p = .147). Subsequent inclusion of NESI in Model 4 (∆R^2^ = .035, p = .096), PHQ-9 in Model 5 (∆R^2^ = .007, p = .442) and GPAQ in Model 6 (∆R^2^ = .001, p = .739) did not significantly improve the model. The final comprehensive model (Model 6), which included all predictors, was not statistically significant (R^2^ = .134, F(6,69) = 1.78, p = .116) and explained only 13.4% of the variance in spatial advantage scores, with no predictors reaching statistical significance (p > .05). Despite this, the age group remained close to significance (p = .056), suggesting a potential age-related trend in spatial processing abilities.

### Supplementary analysis: Age as a continuous predictor

For comparative purposes, we also examined age as a continuous predictor in a simple linear regression model. The model showed only a marginal trend for predicting spatial advantage, F(1, 74) = 2.93, p = .091, R^2^ = .038, with an unstandardized coefficient (β) of −.075 (p = .091). The superior explanatory value of the categorical age group model (∆R^2^ = .023) suggests that differences in spatial processing ability during middle adulthood may reflect a threshold effect between the fifth and sixth decades rather than a strictly linear, year-by-year decline. (See **Table 5** for model details)

**Table 5:** Linear Regression Model for spatial advantage with Age as Continuous Predictor.

Model	R	R^2^	Overall Model Test			
			F	df1	df2	p
1	.195	.0381	2.93	1	74	.091

*Note. Models estimated using a sample size of N = 76.*

### Exploratory nonlinear age effects

Following linear regression analyses, an exploratory polynomial regression analysis was conducted to assess potential nonlinear age relationships with speech-in-noise performance. For the 0° azimuth condition, a second-degree polynomial model revealed a marginally significant quadratic age effect (β = 5.26, p = .049, ΔR^2^ = .042), suggesting possible accelerated decline in later middle age. However, the overall model fit remained non-significant (R^2^ = .052, F (2,73) = 2.02, p = .141). Third-degree (cubic) polynomial models were tested but did not improve model fit (ΔR^2^ = .008, p = .412) or reveal additional significant effects. No significant nonlinear patterns emerged for spatial advantage scores (quadratic p = .828; cubic p = .619), with all polynomial models explaining minimal variance (R^2^ < .04). While these results hint at potential nonlinear age effects in co-located noise perception, the small effect sizes and marginal significance warrant cautious interpretation.

## 4. Discussion

The present study investigated speech perception in noise abilities in middle-aged adults across two age groups (41–50 and 51–60 years), and the influence of cognitive function, education, noise exposure, depression, and physical activity. Our findings revealed notable differences between these age groups, particularly in spatial processing abilities, cognitive function, and education.

### 4.1. Age-related differences in SPIN performance

The current study showed that the 51–60-year-old group performed poorly in SSST-K compared to their 41–50-year-old counterparts. This difference was seen mainly in spatial separation scores and not in the co-located conditions. Recent studies report speech in noise difficulties emerging as early as the 20s, particularly in individuals with tinnitus, elevated [29,36,37] extended high frequency (EHF) thresholds, or substantial noise exposure. This is in contrast with our observation that only adults aged 51–60 years showed reduced spatial advantage. However, our community-based sample comprised self-reported normal hearing adults and likely represents a relatively lower risk group compared to the targeted high-risk cohorts in prior work. Moreover, we did not obtain conventional or EHF audiometry, and thus could not quantify subclinical cochlear or neural deficits that may underlie earlier onset SPIN problems [37]. Our results should therefore be interpreted as characterising age related changes in SPIN among community dwelling, self reported normal hearing middle aged adults, rather than defining the earliest age of SPIN onset. This selective deficit in spatial advantage aligns with the study findings of Gallun et a. [38], which states that age alone can substantially reduce spatial release from masking, independent of hearing loss. This could be due to impaired temporal processing ability starting in midlife [39]. The current results extend these findings by showing that this dissociation emerges clearly in midlife, using an adaptive test paradigm. Replication in the larger sample may be needed to confirm the lack of significant difference at the co-located condition found in our study. The selective deficit in spatial advantage observed in the 51–60-year group, with preserved performance in the co-located condition, is particularly noteworthy but must be interpreted cautiously. While this pattern could reflect central auditory processing changes specific to spatial processing, it could equally result from age-related EHF hearing loss that disproportionally affects binaural spatial processing [40]. Without objective audiometric data, these competing explanations cannot be differentiated.

Previous studies have used standard speech in noise tests [9,15,41] in middle-aged adults and have reported poorer SPIN performance. However, these previous studies have not explored spatial separation using speech in noise tests in middle-aged adults. The current results partially align with the previous literature, which showed age-related SPIN decline after 50 years [12,23]. The difference seen could mainly be due to the method, sample size, and the test materials used. The findings of this study demonstrate that SSST-K is sensitive to subtle, within–middle–aged differences in spatial advantage Importantly, by modelling multiple covariates simultaneously, we also demonstrate that global cognition, rather than noise exposure, depressive symptoms, or physical activity, is the primary predictor of SSST-K performance in this age band, highlighting a clinically actionable link between cognitive screening and SPIN assessment in midlife. In low-resource settings where standard audiometric testing or formal SPIN testing is impractical, a simple administration of the MoCA can serve as a proxy to identify individuals at risk for difficulties with speech in noise. As a secondary observation, the SSST-K demonstrated sensitivity to age-related differences in our middle-aged sample, though formal validation of this property would require dedicated study designs.

### 4.2. Predictors of SPIN performance

Our investigation into potential predictors of SPIN revealed interesting patterns. While significant differences were observed between age groups in spatial release in masking, age as a continuous variable did not emerge as a significant predictor. This suggests that SPIN decline may not occur linearly throughout middle age but may accelerate in the older middle-aged group (51–60 years). Further examining each age point within the middle-aged range is necessary to identify the exact knee point at which SPIN abilities begin to decline more rapidly.

Consistent with previous literature, we observed that cognitive abilities influence SPIN outcomes. Our findings extend this established relationship by demonstrating its persistence in community-dwelling middle-aged adults with self-reported normal hearing, particularly in co-located listening conditions. Using the MoCA to measure general cognition, our findings indicated that cognitive abilities significantly influenced SPIN outcomes, suggesting, consistent with prior models that central auditory and cognitive processes likely interact to support speech perception in noise as individuals age; however, the current study did not include direct measures of central auditory processing, so this interpretation should be considered speculative While MoCA scores emerged as a significant predictor, this global screening tool limits our ability to identify which specific cognitive domains (e.g., working memory, processing speed, inhibitory control) drive this relationship. This relationship aligns with emerging models of auditory-cognitive interactions, where cognitive functions, primarily executive and working memory, compensate for degraded auditory input. [12,19].

This study found that education is co-related to cognitive abilities, which is a highly acceptable hypothesis. However, education was a significant predictor of speech in noise performance. It is generally believed that education increases the cognitive reserve and, in turn, cognition. [14,42]. However, this effect is robust only when low to moderate education is contrasted [43]. Since most of the population in the present study falls in the moderate education category, it may not have influenced cognition or SPIN scores. However, a diverse population with varying levels of education may be needed to confirm the relationship. Also, it is possible that education could indirectly mediate speech in noise score through cognition. Noise exposure showed no significant effects, possibly due to moderate exposure levels of the current sample, recall bias, or sample age potentially obscuring cumulative effects [33]. At the same time, physical activity also had no detectable impact, possibly due to self-reported measures, which lack precision. This contrasts with findings from [44] and [17] and requires further research. Although depression showed no direct link to SPIN performance, its potential interaction with cognitive load [15] highlights the complex interplay of factors shaping auditory processing.

### 4.3. Peripheral hearing considerations

The lack of objective audiometric assessment represents a critical limitation in interpreting our findings. While participants self-reported normal hearing, subclinical hearing loss, loss-particularly in extended high frequencies, is common in middle age and strongly associated with SPIN difficulties. The observed cognitive effects may therefore be due to:

1. Central auditory-cognitive processing differences,
2. Peripheral hearing loss compensated by cognitive resources.
3. A combination of both peripheral and central factors.

Without audiometric data, we cannot affirmatively distinguish between these possibilities. Future research must include a comprehensive hearing assessment, including pure-tone audiometry (0.25–8 kHz), Extended high-frequency testing (9-20kHz), Otoacoustic emissions, and Speech audiometry in quiet.

This limitation is particularly critical for our findings on spatial advantage. Recent research has demonstrated that extended high-frequency sensitivity directly influences binaural spatial release from masking [40], even when EHF cues are not explicitly present in the speech signal. Monson and Trine (2023) [45] further documented that EHF hearing loss predicts speech-in-noise performance independently of conventional audiometric thresholds. Given that Stelmachowicz et al. [24] identified the 40–59-year age range precisely our study population as exhibiting the largest age-related EHF threshold shifts, the observed group difference in spatial advantage (41–50 vs. 51–60 years) could reflect accumulated EHF hearing loss rather than, or in addition to, central auditory processing decline. This alternative explanation cannot be ruled out without objective EHF assessment.

### 4.4. Implications for clinical practice

The findings indicate that, where available, clinical assessments for middle-aged adults with listening difficulties should include not only traditional speech-in-noise tests but also measures of spatial hearing (e.g., SSST-K) as a proxy to identify early auditory processing issues. Additionally, incorporating cognitive screenings focused on executive function and working memory into routine audiological evaluations is essential due to the strong link between cognitive function and speech perception.

### 4.5. Limitations and future directions

This study has several significant limitations. A significant limitation of this study is the reliance on self-reported hearing status without objective audiometric verification. The absence of pure tone audiometry (PTA), particularly extended high frequency (EHF) testing above 8 kHz, means we cannot definitively rule out subtle peripheral hearing loss that may have confounded our results. Research has shown that middle-aged adults frequently exhibited threshold shifts in EHF ranges that can significantly impact speech in noise perception [24,45], even when conventional audiometry (≤8 kHz) appears normal. Therefore, what we attributed to cognitive factors may partially reflect undetected peripheral auditory decline. Additionally, the absence of audiometric data precluded sensitivity analyses that could have tested the robustness of our findings across different model specifications, such as comparing results with and without hearing threshold covariates.

The cross-sectional design restricts causal conclusions and the understanding of auditory changes across midlife. Reliance on self-report measures for noise exposure (without objective verification via dosimetry or work records) and physical activity introduces potential recall bias. Moreover, we were unable to conduct meaningful sensitivity analyses, such as stratifying by noise exposure levels or comparing subgroups with documented occupational noise exposure, because detailed objective verification data (e.g., dosimetry, work history records) were not available. The cognitive assessment used (MoCA) provides a global screening score and does not assess specific cognitive domains (e.g., working memory, executive function, processing speed) known to differentially impact speech-in-noise perception. Our findings, therefore, relate to general cognitive function rather than specific cognitive mechanisms. Additionally, the use of snowball sampling limits generalizability to the broader middle-aged population, as this method may have selected for more educated, socially connected, or health-conscious individuals. These factors highlight the need for future longitudinal studies using comprehensive cognitive evaluations and objective lifestyle measures across a wider demographic.

## 5. Conclusion

The study demonstrates that speech perception in noise difficulties are observable in middle age, with general cognitive function emerging as a significant correlation of SPIN performance in our sample of adults with self-reported normal hearing. However, the absence of objective audiometric verification- particularly extended high frequency testing prevents definitive conclusions about whether these effects are truly independent of peripheral hearing status. These findings underscore the need for comprehensive hearing assessment protocols in future research examining SPIN abilities in middle-aged populations.

## Supporting information

S1 File Supplementary Regression Diagnostics. (DOCX)

## References

[1] Humes LE. Factors underlying the speech-recognition performance of elderly hearing-aid wearers. J Acoust Soc Am. 2002;112(3 Pt 1):1112–32. doi: 10.1121/1.1499132 12243159

[2] Humes LE, Roberts L. Speech-recognition difficulties of the hearing-impaired elderly: the contributions of audibility. J Speech Hear Res. 1990;33(4):726–35. doi: 10.1044/jshr.3304.726 2273886

[3] Jerger J, Jerger S, Pirozzolo F. Correlational analysis of speech audiometric scores, hearing loss, age, and cognitive abilities in the elderly. Correlational analysis of speech audiometry in elderly. 1991. 10.1097/00003446-199104000-00004 2065833

[4] Dubno JR, Horwitz AR, Ahlstrom JB. Benefit of modulated maskers for speech recognition by younger and older adults with normal hearing. J Acoust Soc Am. 2002;111(6):2897–907. doi: 10.1121/1.1480421 12083223

[5] Helfer KS, Freyman RL. Stimulus and listener factors affecting age-related changes in competing speech perception. J Acoust Soc Am. 2014;136(2):748–59. doi: 10.1121/1.4887463 25096109 PMC4187459

[6] Dawes P, Fortnum H, Moore DR, Emsley R, Norman P, Cruickshanks K, et al. Hearing in middle age: a population snapshot of 40- to 69-year olds in the United Kingdom. Ear Hear. 2014;35(3):e44-51. doi: 10.1097/AUD.0000000000000010 24518430 PMC4264521

[7] Demeester K, Topsakal V, Hendrickx J-J, Fransen E, van Laer L, Van Camp G, et al. Hearing disability measured by the speech, spatial, and qualities of hearing scale in clinically normal-hearing and hearing-impaired middle-aged persons, and disability screening by means of a reduced SSQ (the SSQ5). Ear Hear. 2012;33(5):615–6. doi: 10.1097/AUD.0b013e31824e0ba7 22568994

[8] Nachtegaal J, Smit JH, Smits C, Bezemer PD, van Beek JHM, Festen JM, et al. The association between hearing status and psychosocial health before the age of 70 years: results from an internet-based national survey on hearing. Ear Hear. 2009;30(3):302–12. doi: 10.1097/AUD.0b013e31819c6e01 19322094

[9] McFarlane KA, Sanchez JT. Effects of temporal processing on speech-in-noise perception in middle-aged adults. Biology (Basel). 2024;13(6). 10.3390/biology13060371 PMC11200514 38927251

[10] Lee JY, Lee JT, Heo HJ, Choi CH, Choi SH, Lee K. Speech recognition in real-life background noise by young and middle-aged adults with normal hearing. J Audiol Otol. 2015;19(1):39–44. 26185790 10.7874/jao.2015.19.1.39 PMC4491949

[11] Guest H, Munro KJ, Prendergast G, Millman RE, Plack CJ. Impaired speech perception in noise with a normal audiogram: no evidence for cochlear synaptopathy and no relation to lifetime noise exposure. Hear Res. 2018;364:142–51. 29680183 10.1016/j.heares.2018.03.008 PMC5993872

[12] Moore DR, Edmondson-Jones M, Dawes P, Fortnum H, McCormack A, Pierzycki RH. Relation between speech-in-noise threshold, hearing loss and cognition from 40–69 years of age. PLoS One. 2014;9(9):e107720. doi: 10.1371/journal.pone.0107720 PMC4168235 25229622

[13] Helfer KS, Freyman RL. Aging and speech-on-speech masking. Ear Hear. 2008;29(1):87–98. doi: 10.1097/AUD.0b013e31815d638b 18091104 PMC2987598

[14] Wilson RS, Yu L, Lamar M, Schneider JA, Boyle PA, Bennett DA. Education and cognitive reserve in old age. Neurology. 2019;92(10). 10.1212/WNL.0000000000007036 PMC6442015 30728309

[15] Xie Z, Zinszer BD, Riggs M, Beevers CG, Chandrasekaran B. Impact of depression on speech perception in noise. PLoS One. 2019;14(8):e0220928. doi: 10.1371/journal.pone.0220928 31415624 PMC6695097

[16] Jiang K, Armstrong NM, Agrawal Y, Gross AL, Schrack JA, Lin FR, et al. Associations of audiometric hearing and speech-in-noise performance with cognitive decline among older adults: The Baltimore Longitudinal Study of Aging (BLSA). Front Neurol. 2022;13. 10.3389/fneur.2022.1029851 PMC9784219 36570462

[17] Gopinath M, Bhat JS, Ranjan R. Effect of sudarshankriya yoga on some auditory processing abilities and speech perception in noise among middle aged adults. Indian J Public Health Res Development. 2018;9(12):213–8.

[18] Hermon AM, Ganapathy K, Palaniswamy HP, Muthu ANP. Development and validation of the spatial separation sentence test in Kannada. J Audiol Otol. 2024;28(3):228–35. 38685832 10.7874/jao.2023.00325 PMC11273185

[19] Akeroyd MA. Are individual differences in speech reception related to individual differences in cognitive ability? A survey of twenty experimental studies with normal and hearing-impaired adults. Int J Audiol. 2008;47 Suppl 2:S53-71. doi: 10.1080/14992020802301142 19012113

[20] Glyde H, Hickson L, Cameron S, Dillon H. Problems hearing in noise in older adults. Trends Amplif. 2011;15(3):116–26. 22072599 10.1177/1084713811424885 PMC4040826

[21] Gopinath M, Bhat JS, Ranjan R. Effect of sudarshankriya yoga on some auditory processing abilities and speech perception in noise among middle aged adults. Ind J Publ Health Res Develop. 2018;9(12):213. doi: 10.5958/0976-5506.2018.01835.1

[22] Guest H, Munro KJ, Prendergast G, Millman RE, Plack CJ. Impaired speech perception in noise with a normal audiogram: No evidence for cochlear synaptopathy and no relation to lifetime noise exposure. Hear Res. 2018;364:142–51. doi: 10.1016/j.heares.2018.03.008 29680183 PMC5993872

[23] Mathew S, Kumar S, Jain C Banumathi, . Effect of age on speech perception in noise abilities across different stimulus. Indian J Otolaryngol Head Neck Surg. 2023;75(4):3718–24. doi: 10.1007/s12070-023-04084-7 37974785 PMC10646145

[24] Stelmachowicz PG, Beauchaine KA, Kalberer A, Jesteadt W. Normative thresholds in the 8- to 20-kHz range as a function of age. J Acoust Soc Am. 1989;86(4):1384–91. 2808912 10.1121/1.398698

[25] Oosterloo BC, Homans NC, Baatenburg de Jong RJ, Ikram MA, Nagtegaal AP, Goedegebure A. Assessing hearing loss in older adults with a single question and person characteristics; Comparison with pure tone audiometry in the Rotterdam Study. PLoS One. 2020;15(1):e0228349. doi: 10.1371/journal.pone.0228349 31986178 PMC6984733

[26] Humes LE. U.S. population data on hearing loss, trouble hearing, and hearing-device use in adults: national health and nutrition examination survey, 2011–12, 2015–16, and 2017–20. Trends Hear. 2023;27. 10.1177/23312165231160978 PMC10084570 37016920

[27] Nasreddine ZS, Phillips NA, Bédirian V, Charbonneau S, Whitehead V, Collin I, et al. The Montreal Cognitive Assessment, MoCA: a brief screening tool for mild cognitive impairment. J Am Geriatr Soc. 2005;53(4):695–9. doi: 10.1111/j.1532-5415.2005.53221.x 15817019

[28] Kaul S, Paplikar A, Varghese F, Alladi S, Sharma M, Dhaliwal RS. MoCA in five Indian languages: A brief screening tool to diagnose dementia and MCI in a linguistically diverse setting. Int J Geriatr Psychiatry. 2022;37(10). 10.1002/gps.5808 36069187

[29] Gilles A, Schlee W, Rabau S, Wouters K, Fransen E, Van de Heyning P. Decreased speech-in-noise understanding in young adults with tinnitus. Front Neurosci. 2016;10. 10.3389/fnins.2016.00288 PMC4923253 27445661

[30] Kroenke K, Spitzer RL, Williams JBW. The PHQ-9. J Gen Intern Med. 2001;16(9). 10.1046/j.1525-1497.2001.016009606.x PMC1495268 11556941

[31] Kochhar PH, Rajadhyaksha SS, Suvarna VR. Translation and validation of brief patient health questionnaire against DSM IV as a tool to diagnose major depressive disorder in Indian patients. J Postgrad Med. 2007;53(2):102–7. doi: 10.4103/0022-3859.32209 17495375

[32] Guest H, Dewey RS, Plack CJ, Couth S, Prendergast G, Bakay W. The noise exposure structured interview (NESI): an instrument for the comprehensive estimation of lifetime noise exposure. Trends Hear. 2018;22. 10.1177/2331216518803213 PMC6176535 30295145

[33] Armstrong T, Bull F. Development of the world health organization global physical activity questionnaire (GPAQ). J Public Health (Bangkok). 2006;14(2):66–70.

[34] Bull FC, Maslin TS, Armstrong T. Global physical activity questionnaire (GPAQ): nine country reliability and validity study. J Phys Act Health. 2009;6(6):790–804. doi: 10.1123/jpah.6.6.790 20101923

[35] Rönnberg J, Rudner M, Lunner T, Zekveld AA. When cognition kicks in: working memory and speech understanding in noise. Noise Health. 2010;12(49):263–9. doi: 10.4103/1463-1741.70505 20871181

[36] Zink ME, Zhen L, McHaney JR, Klara J, Yurasits K, Cancel VE, et al. Increased listening effort and cochlear neural degeneration underlie speech-in-noise deficits in normal-hearing middle-aged adults. Elife. 2025;13. 10.7554/eLife.102823 PMC12283073 40694041

[37] Balan JR, Mishra SK, Rodrigo H. Extended high-frequency hearing and suprathreshold neural synchrony in the auditory brainstem. J Acoust Soc Am. 2025;157(3):1577–86. doi: 10.1121/10.0036054 40035573

[38] Gallun FJ, Diedesch AC, Kampel SD, Jakien KM. Independent impacts of age and hearing loss on spatial release in a complex auditory environment. Front Neurosci. 2013;7:252. doi: 10.3389/fnins.2013.00252 24391535 PMC3870327

[39] McFarlane KA, Sanchez JT. Effects of temporal processing on speech-in-noise perception in middle-aged adults. Biol (Basel). 2024;13(6). 10.3390/biology13060371 PMC11200514 38927251

[40] Saxena U, Mishra SK, Rodrigo H, Choudhury M. Functional consequences of extended high frequency hearing impairment: Evidence from the speech, spatial, and qualities of hearing scale. J Acoust Soc Am. 2022;152(5):2946. doi: 10.1121/10.0015200 36456291

[41] Murphy CFB, Rabelo CM, Silagi ML, Mansur LL, Schochat E. Impact of educational level on performance on auditory processing tests. Front Neurosci. 2016;10:97. doi: 10.3389/fnins.2016.00097 27013958 PMC4785234

[42] Murphy CFB, Rabelo CM, Silagi ML, Mansur LL, Schochat E. Impact of Educational Level on Performance on Auditory Processing Tests. Front Neurosci. 2016;10. 27013958 10.3389/fnins.2016.00097 PMC4785234

[43] van Hooren SAH, Valentijn AM, Bosma H, Ponds RWHM, van Boxtel MPJ, Jolles J. Cognitive functioning in healthy older adults aged 64–81: a cohort study into the effects of age, sex, and education. Aging Neuropsychology Cognition. 2007;14(1):40–54. 10.1080/138255890969483 17164189

[44] Kearns L, Rich A, Pita N, Okada K. Spin: the effects of acute exercise on speech perception. PBS. 2019;8(3):67. doi: 10.11648/j.pbs.20190803.12

[45] Monson BB, Trine A. Extending the high-frequency bandwidth and predicting speech-in-noise recognition: building on the work of Pat Stelmachowicz. Semin Hear. 2023;44(S 01):S64-74. 10.1055/s-0043-1764133 PMC10033195 36970650

