# Supplementary material for: Influence of age on speech-in-noise and spatial processing abilities in middle-aged adults
Source: PLoS One. 2026 Jan 29;21(1):e0341169. doi: 10.1371/journal.pone.0341169 (PMC12854412; doi:10.1371/journal.pone.0341169)
Supplement: S1 File — (DOCX) [file pone.0341169.s001.docx]

**Suppl Table 1.** Multicollinearity Diagnostics for 0° Azimuth Condition Regression Models.

| Model | Predictor | VIF | Tolerance |
| --- | --- | --- | --- |
| 1 | Age Group | 1.00 | 1.00 |
| 2 | Age Group | 1.10 | .911 |
|  | MoCA | 1.10 | .911 |
| 3 | Age Group | 1.13 | .885 |
|  | MoCA | 1.56 | .639 |
|  | Education | 1.57 | .635 |
| 4 | Age Group | 1.14 | .880 |
|  | MoCA | 1.60 | .625 |
|  | Education | 1.58 | .633 |
|  | NESI | 1.08 | .925 |
| 5 | Age Group | 1.17 | .857 |
|  | MoCA | 1.76 | .568 |
|  | Education | 1.62 | .617 |
|  | NESI | 1.08 | .925 |
|  | PHQ-9 | 1.12 | .895 |
| 6 | Age Group | 1.18 | .846 |
|  | MoCA | 1.76 | .568 |
|  | Education | 1.63 | .612 |
|  | NESI | 1.11 | .902 |
|  | PHQ-9 | 1.12 | .895 |
|  | GPAQ | 1.08 | .929 |
| *Note. For both tables: VIF = Variance Inflation Factor. All VIF values are below the conservative threshold of 2.5, indicating no concerning multicollinearity in any models. Tolerance = 1/VIF.* | | | |

**Suppl Table 2.** Multicollinearity Diagnostics for Spatial Advantage Regression Models.

| Model | Predictor | VIF | Tolerance |
| --- | --- | --- | --- |
| 1 | Age Group | 1.00 | 1.00 |
| 2 | Age Group | 1.10 | .911 |
|  | MoCA | 1.10 | .911 |
| 3 | Age Group | 1.13 | .885 |
|  | MoCA | 1.56 | .639 |
|  | Education | 1.57 | .635 |
| 4 | Age Group | 1.14 | .880 |
|  | MoCA | 1.60 | .625 |
|  | Education | 1.58 | .633 |
|  | NESI | 1.08 | .925 |
| 5 | Age Group | 1.17 | .857 |
|  | MoCA | 1.76 | .568 |
|  | Education | 1.62 | .617 |
|  | NESI | 1.08 | .925 |
|  | PHQ-9 | 1.12 | .895 |
| 6 | Age Group | 1.18 | .846 |
|  | MoCA | 1.76 | .568 |
|  | Education | 1.63 | .612 |
|  | NESI | 1.11 | .902 |
|  | PHQ-9 | 1.12 | .895 |
|  | GPAQ | 1.08 | .929 |
| *Note. For both tables: VIF = Variance Inflation Factor. All VIF values are below the conservative threshold of 2.5, indicating no concerning multicollinearity in any models. Tolerance = 1/VIF.* | | | |

***Linear regression Assumptions***

*Collinearity statistics revealed acceptable VIF values (all VIF < 2.0), indicating no multicollinearity. Shapiro-Wilk test of residuals indicated marginal deviation from normality (W = 0.966, p = .010); however, the Q-Q plot of residuals revealed that residuals were approximately normally distributed, with minor deviations at the extremes. The residual plot demonstrated homogeneous variance across fitted values, confirming the homoscedasticity assumption. Cook's distance analysis identified no influential outliers. The maximum Cook's distance was 0.0975 (well below the conventional threshold of 1.0), with a median of 0.0175 and a mean of 0.00454, indicating that all observations had minimal and comparable influence on the regression results. These findings align with established guidance that linear regression is robust to minor deviations from normality.*

***Suppl Figure 1: Q-Q Plot***


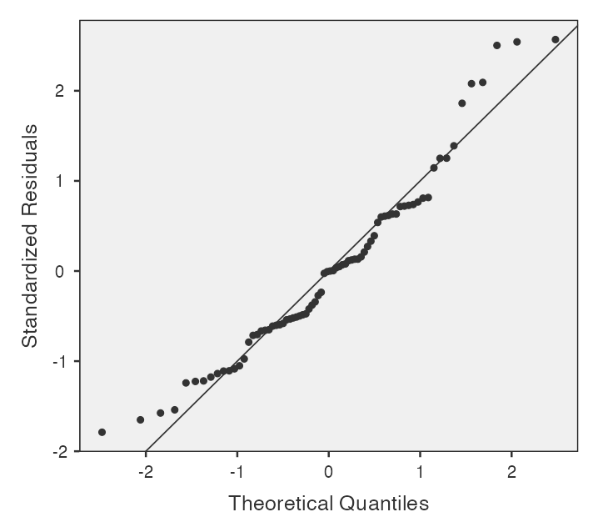


| ***Suppl Table 3: Cook's Distance*** | | | | |
| --- | --- | --- | --- | --- |
|  | | | ***Range*** | |
| ***Mean*** | ***Median*** | ***SD*** | ***Min*** | ***Max*** |
| *0.0118* | *0.00454* | *0.0175* | *4.33* | *0.0975* |
